# Epidemiology and renal injury following 2-methyl-4-chlorophenoxyacetic acid (MCPA) poisoning

**DOI:** 10.1038/s41598-022-25313-z

**Published:** 2022-12-19

**Authors:** Thilini M. Wijerathna, Nicholas A. Buckley, Indika B. Gawarammana, Jacques Raubenheimer, Seyed Shahmy, Umesh Chathuranga, Chathura Palangasinghe, Fathima Shihana, Fahim Mohamed

**Affiliations:** 1 South Asian Clinical Toxicology Research Collaboration, Faculty of Medicine, University of Peradeniya, Peradeniya, Sri Lanka; 2 Department of Biosystems Technology, Faculty of Technology, General Sir John Kotelawala Defence University, Ratmalana, Sri Lanka; 3 Clinical Pharmacology and Toxicology Research Group, Faculty of Medicine and Health, Biomedical Informatics and Digital Health, The University of Sydney, Sydney, NSW 2006 Australia; 4 Department of Medicine, Faculty of Medicine, University of Peradeniya, Peradeniya, Sri Lanka; 5 National Science and Technology Commission of Sri Lanka, Colombo, Sri Lanka; 6 Centenary Institute of Cancer Medicine & Cell Biology, The University of Sydney, Camperdown, NSW Australia; 7 Department of Pharmacy, Faculty of Allied Health Science, University of Peradeniya, Peradeniya, Sri Lanka; 8 The Edith Collins Centre (Translational Research in Alcohol Drugs and Toxicology), Sydney Local Health District, & The University of Sydney, Faculty of Medicine and Health, Department of Pharmacology, Sydney Pharmacy School, The University of Sydney, Sydney, Australia; 9 Australian Kidney Biomarker Reference Laboratory, Department of Nephrology, Prince of Wales Hospital and Clinical School, University of New South Wales, Sydney, Australia

**Keywords:** Biomarkers, Medical research, Nephrology

## Abstract

2-Methyl-4-chlorophenoxyacetic acid (MCPA) is a widely used chlorophenoxy herbicide. MCPA poisoning causes mitochondrial dysfunction, which can lead to kidney injury and death. The objective of this study is to describe the epidemiology, case fatality and extent of renal injury in a large cohort of MCPA self-poisonings. The study consists of two parts: (1) A report of epidemiological data and clinical outcomes in MCPA poisoned patients in Sri Lanka between 2002 and 2019; (2) Evaluation of acute kidney injury (AKI) using renal biomarkers in a subset from this cohort. Serum creatinine (sCr) and biomarkers were measured soon after hospitalization (2 [IQR 1–3] h) and at different time intervals. We measured serum biomarkers: sCr, cystatin C (sCysC), creatine kinase (CK), and urinary biomarkers: creatinine, kidney injury molecule-1 (KIM-1), clusterin, albumin, beta-2-microglobulin (β2M), cystatin C, neutrophil gelatinase-associated lipocalin (NGAL), osteopontin (OPN), trefoil factor 3 (TFF3) and cytochrome *C* (CytoC). Kidney Disease Improving Global Outcomes (KDIGO) criteria was used to define acute kidney injury (AKI). There were 1653 patients; 65% were male. The median time from ingestion to examination was 3:54 (IQR 2:19–6:57) h. The overall case-fatality rate was 5.3%. Patients who died were older (42 [IQR 33.5–54] vs 27 [IQR 20–37] for survivors). The median estimated amount of MCPA ingested by patients who died was also greater (88 [IQR 34–200] vs. 30 [IQR 15–63] ml in survivors). Moderate to severe AKI (AKI2/3) was uncommon (6/59 patients in the biomarker study had KDIGO stage 2 or 3). Most patients in AKI2/3 group with increased sCr were older (median age 35 years [IQR 27–41]) compared to No AKI (23 years (19–29) years) or AKI1 (26 years (21–40) years) group who had no or mild increase in sCr. These patients had no pre-existing kidney diseases. In these patients, serum creatinine (maximum medium concentration; 1.12 [IQR 0.93–1.67] mg/dl) and CK (maximum medium concentration; 284 [IQR 94–428] U/l) were increased but sCysC (maximum medium concentration; 0.79 [IQR 0.68–0.81] mg/l) remained in the normal range within 72 h. All urinary biomarkers performed poorly in diagnosing AKI (area under the receiver operating characteristic curve < 0.68). The higher numbers of men with MCPA poisoning likely reflects greater occupational access to pesticides. Fatal outcome and higher ingested dose were more common in the elderly. Significant AKI with tubular injury biomarkers was uncommon. Most people with raised sCr were older and appeared to have no pre-existing kidney disease.

## Introduction

MCPA (2-methyl-4-chlorophenoxyacetic acid) is a widely used herbicide. Toxic effects of MCPA include cellular dysfunction by disruption of lipid membranes, interference with metabolic pathways involving acetyl coenzyme A, and uncoupling of oxidative phosphorylation^1^.

The clinical effects and laboratory features have been reported in fine detail in only a few small case series^1,2^. A much larger prospective cohort study reported 8 deaths in 181 cases from Sri Lanka with some limited clinical data. These deaths mostly occurred between 24 and 48 h post poisoning. This study did not have laboratory data on kidney injury, and also did not identify any factors associated with a fatal outcome^3^.

Nephrotoxicity following MCPA poisoning is believed to be an important predictor of severe toxicity or death^3^. Although renal elimination is an important route of elimination of MCPA^4^, renal injury after MCPA poisoning has not been studied extensively. The nature and sites of the kidney damage are also unknown.

Therefore, in this report we describe epidemiological data from a cohort study, with the aim of defining factors associated with a fatal outcome. We also evaluated renal toxicity in a subset of patients from this cohort using functional and structural damage renal biomarkers.

## Materials and methods

### Study design and patient recruitment

The study was approved by the Human Research Ethics Committees of the Faculty of Medicine, University of Peradeniya, Peradeniya, Sri Lanka and the University of New South Wales, Australia. This was part of a large ongoing multicentre prospective cohort study on acute intentional self-poisoning in Sri Lanka which has recruited more than 80,000 patients (> 29,000 ingesting agrochemicals) prospectively over 18 years^5^. Patients received basic standard patient care during hospitalization (medical and nursing observations, intravenous fluids). We report data on MCPA poisoning collected prospectively from consenting patients who presented to 9 hospitals (Peradeniya, Nuwara-Eliya, Anuradhapura, Polonnaruwa, Galle, Matara, Kurunegala, Chilaw and Marawila) located in Central, North Central, Southern and North Western Province of Sri Lanka over a period of 18 years (2002–2019). Exclusion criteria for the cohort were age < 15 years, confirmed pregnancy, mixed overdose and late presenters (more than 24 h after ingestion). The need for informed consent was waived by the ethics review committee for this cohort study but informed written consent was obtained from patients or an accompanying relative on hospital admission to enroll in renal biomarker study. All procedures performed the study involving human participants were in accordance with the ethical standards of the institutional research committee and with the 1964 Helsinki declaration and comparable ethical standards.

The study consists of two parts:

1. *A report on epidemiologic and outcome data*
  All consenting MCPA self-poisoning patients (based on patient history, label examination, identification based on pesticide bottle brought by patients/relatives, transfer notes from the peripheral hospital, assay confirmation in some patients) admitted to SACTRC study hospitals between April 2002 and December 2019 were included in the analysis. Epidemiologic, demographic and clinical data were recorded on a study-specific data sheet, supplemented by hospital medical records.
2. *Evaluation of biomarkers of acute kidney injury *(*AKI*)
  Of this large prospective cohort, a subset of symptomatic patients (as documented by the physician between January 2011 and July 2014) were selected to evaluate renal biomarkers (n = 59). Patients who provided ≥ 2 blood and urine samples during their hospital stay (at least till 72 h post-ingestion) then daily until discharge or death including a minimum one sample within 24 h post-ingestion (median no. of samples collected 4^3–5^) were included to this subset (n = 59). There were no patients with known comorbid conditions based on their medical history. Additional samples were collected 1 month and/or 2–7 months after discharge from the hospital to explore the long-term effects of acute MCPA overdose on renal function. However, all the patients follow up data were not available.

Kidney Disease Improving Global Outcomes (KDIGO) criteria was used to define acute kidney injury (AKI). Acute kidney injury stage 1 is defined as an increase in sCr by ≥ 0.3 mg/dl within 48 h or an increase in sCr to 1.5–2-fold from baseline, which is presumed to have occurred within the prior 7 days. AKI stage 2 is defined as increase in sCr ≥ 2.0 but < 3 times from baseline while AKI stage 3 is defined as ≥ 3.0 times baseline or increase in sCr to ≥ 4.0 mg/dl (≥ 354 μmol/l) or initiation of renal replacement therapy^6^. The baseline sCr was defined as the lowest sCr > 0.4 mg/dl on admission or during 1–7 months post discharge^7–10^. Patients were grouped into no evidence of AKI (No AKI), AKI stage 1 or AKI stage 2/3.

### Laboratory assays for biomarkers

We quantified serum and urine biomarkers from the samples collected during 4, 8, 16, 24 and 72 h post-ingestion and 1–7 months of follow up. The biomarkers measured were: creatinine (sCr) (Jaffe kinetic method), serum cystatin C (sCysC) (Immunoturbidimetry method), creatine kinase (CK) (Dry chemistry analyzer, rapid test) and serum Total Protein (sTP) (refractometry method). Enzyme-linked immunosorbent assays (ELISA) were used for urinary kidney injury molecule-1 (uKIM-1), clusterin (uClu), and Cytochrome *C* (uCytoC). A Bio-PlexPro™RBM human kidney toxicity assay panel was used for albumin (uAlb), beta-2-microglobulin (uβ2M), urine cystatin C (uCysC), neutrophil gelatinase associated lipocalin (NGAL), osteopontin (uOPN) and trefoil factor 3 (uTFF 3) as explained in more detail in our previous report^7,9^. All urinary biomarker levels were normalized to urinary creatinine.

### Statistical analysis

All continuous variables are expressed as medians and interquartile ranges (IQRs). Continuous data were compared using Mann–Whitney *U* test, Receiver Operating Characteristics Curve (ROC) Test and correlated with Spearman correlation. The categorical data were compared using Fisher’s exact test and Clopper–Pearson (C–P) exact confidence intervals are provided for proportions. The time courses of each biomarker are shown using spaghetti plots (Level of significance < 0.05, Software used; GraphPad Prism 7).

## Results

### Basic demographic characteristics of survivors and non-survivors

The cohort contained 1625 cases where MCPA was recorded on admission at hospital, and MCPA was detected in the sample of an additional 28 cases who had taken unknown substances or unknown pesticides, for a total of 1653 MCPA cases, of which most (N = 1079, 65.3%) were male. The median time from ingestion to examination was 3:54 (N = 1573, IQR 2:19–6:57) h, and 75% were transferred from peripheral hospitals. Eighty-eight died with a case fatality of 5.3% (95% C–P CI 4.3–6.5%). Patients who died were older: 42 years [IQR 33.5–54] vs 27 years [IQR 20–37] for survivors (95% CIs for medians: 38–48 and 26–28 respectively). The median time from ingestion to death was 19 [IQR 7–35] h. The median amount of MCPA ingested by patients who died was also greater (88 ml [IQR 34–200] vs. 30 ml [IQR 15–63] in survivors). The median Glasgow Coma Scale (GCS) scores at admission for survivors and death category were 15 (15–15) and 12 (7.5–15) respectively (Table 1). Clinical data were limited, but an initial tachycardia was associated with a fatal outcome. Other clinical features (blood pressure, respiratory rate) did not predict death at the time of admission (Table 1). There was no significant difference noted between survivors (34%) and fatalities (24%) in terms of occurrence of AKI (of 59 patients who had on admission sCr values).

**Table 1:** Demographic and clinical data at the time of admission in the cohort of survivors and fatalities.

Parameter	Survivors (n = 1565)	Fatalities (n = 88)	p value
Time to admission—h (IQR)	4 (2–7)	4 (3–8)	0.9060
Estimated amount ingested (ml)	30 (15–63)	88 (34–200)	< 0.0001
Male—n (%)	1057 (65%)	58 (76%)	0.048
Age in years (IQR)	27 (20–37)	42 (33.5–54)	< 0.0001
Systolic BP—mmHg (IQR)	120 (110–120)	110 (100–120)	0.017
Diastolic BP—mmHg (IQR)	80 (70–80)	70 (60–80)	0.0006
Heart rate—beats/minute (IQR)	81 (78–92)	93 (84–110)	< 0.0001
Respiratory rate—per minute (IQR)	20 (18–20)	20 (18–26)	0.0034
GCS score at admission	15 (15–15)	12 (7.5–15)	< 0.0001

Data expressed as number of patients (n), percentage (%) or median (lower quartile − upper quartile). Two groups were compared using Mann–Whitney *U* test or Fisher Exact test. All the clinical data reported here were taken during the admission to the study hospitals.

### Acute agricultural chemical poisoning and epidemiological trend of common herbicides

**Figure 1:**
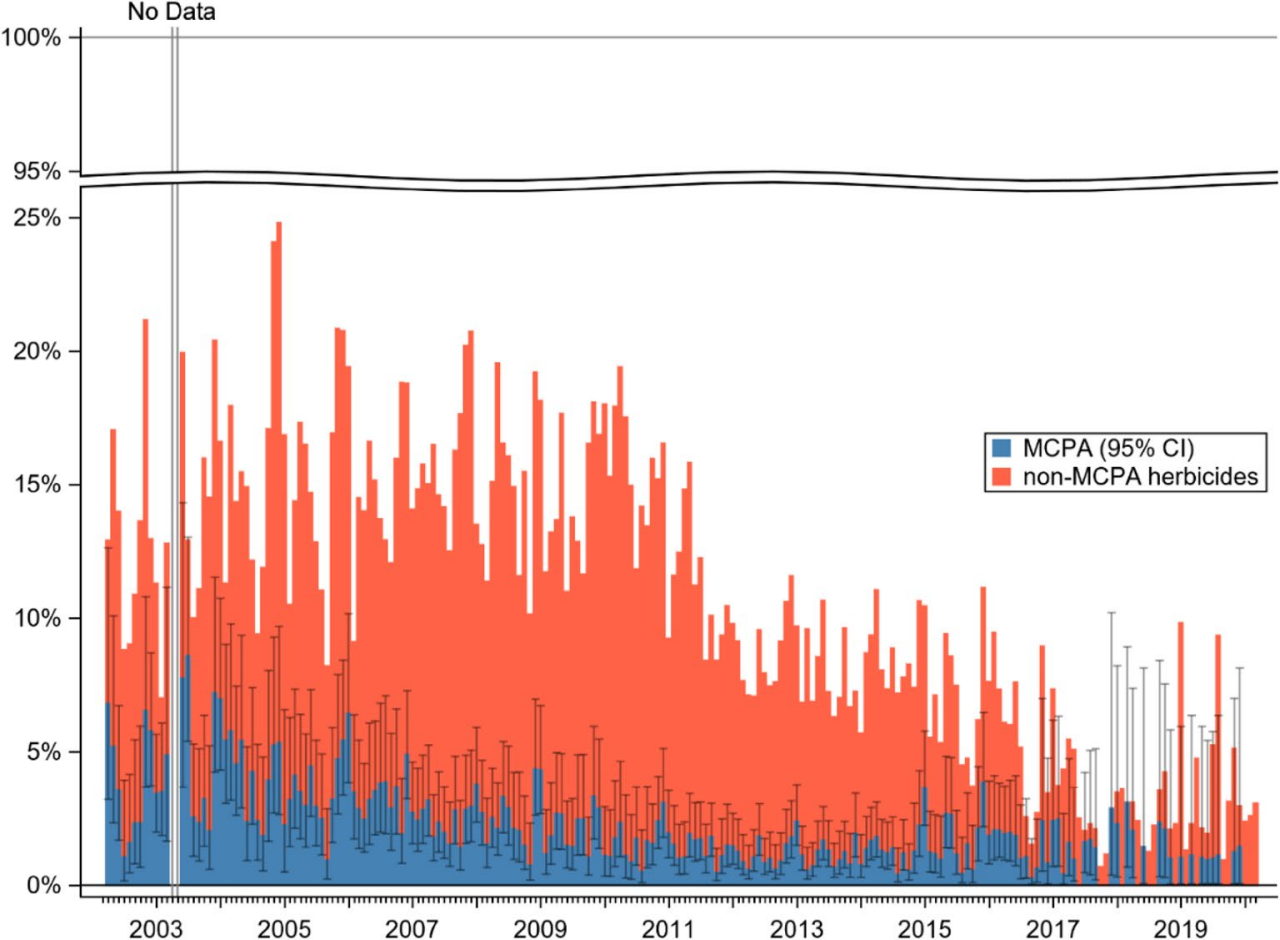
Herbicide self-poisoning (MCPA vs all other herbicides) compared to all other poisoning over time.

There were 8872 (877 deaths) cases of herbicide self-poisoning presentations to SACTRC study hospitals over the 18-year period. A marked decrease in the proportion of herbicide ingestions relative to all poisonings can be seen over the study period (Fig. 1), from a high of 25% in December 2004, to 5.2% in November 2019, and lower in the following months.

**Figure 2:**
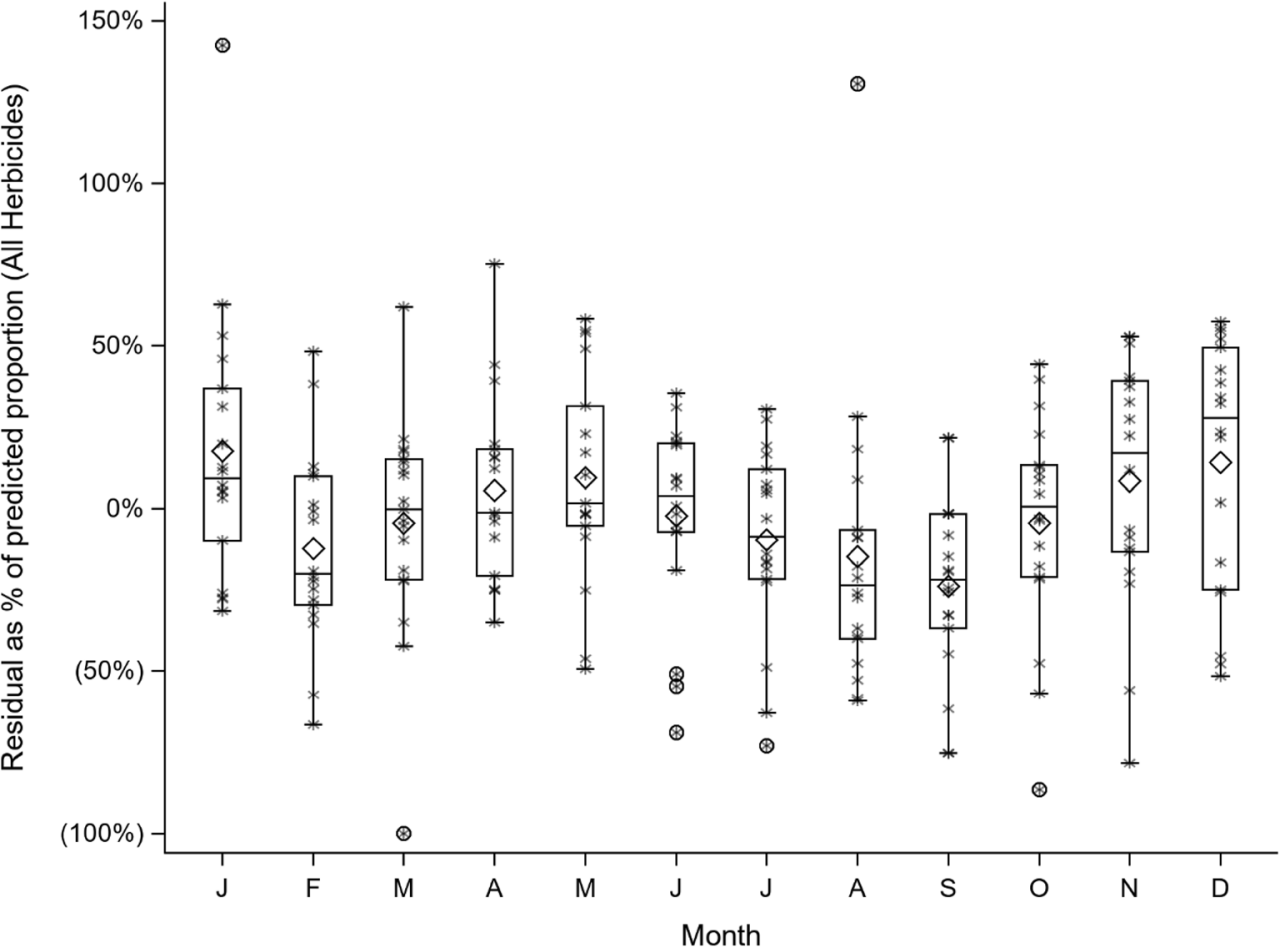
Monthly deviation from yearly predicted values of percentage of herbicide self-poisonings.

A distinct seasonal pattern is also discernible in Fig. 1. As such in, a linear regression modelling, the percentage of herbicide cases against the study year was computed. Then, the residuals for each month of every year were calculated against the predicted value for that year, and to account for the declining proportion of herbicide cases over time, expressed as percentage of the predicted value, as shown in Fig. 2, where a distinct increase in the proportion of herbicide cases is visible during the planting seasons of March through June and September through December.

### Pattern of renal toxicity and renal biomarker levels

**Figure 3:**
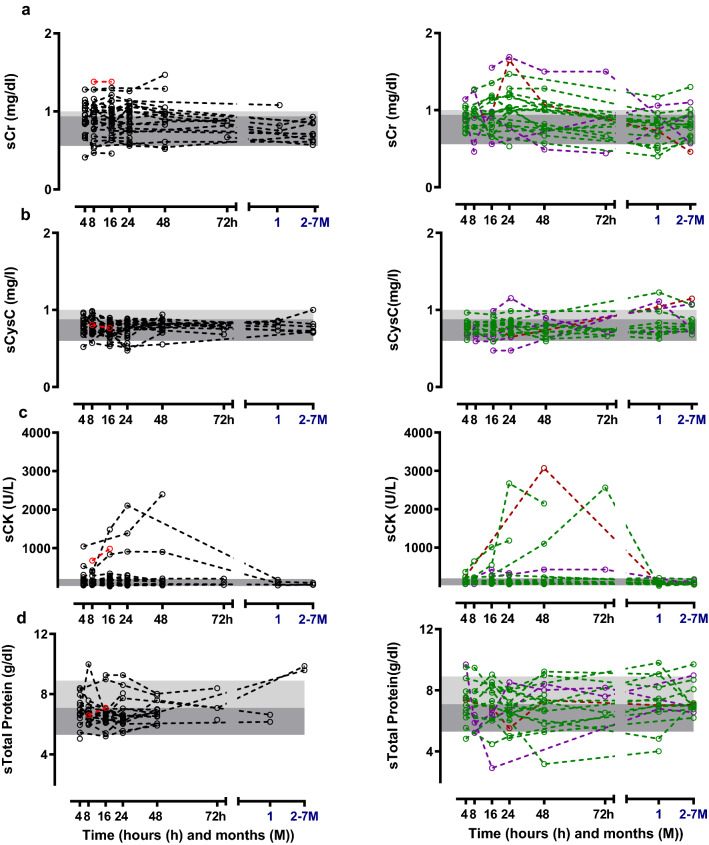

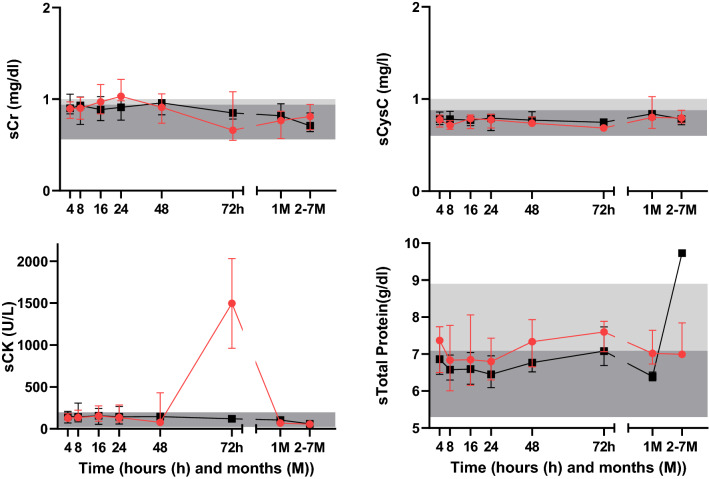
(**A**) Serum biomarker concentration profiles. Individual patient’s biomarker concentrations of serum (**a**) creatinine, (**b**) cystatin C, (**c**) CK and (**d**) Total Protein are shown [black dotted line—No AKI (alive (n = 34)), red dotted line—death (n = 1), green dotted line—AKIN1 (alive (n = 18)), purple dotted line—AKIN2 (alive (n = 5)) and brown dotted line—AKIN3 (alive (n = 1))]. The grey color shaded area illustrates 5th–75th percentile and light grey color shaded area indicates 75th–95th percentile of the normal range based on measurements in healthy individuals. (**B**) Changes in serum biomarker concentrations. Median and inter quartile ranges (IQRs) of the biomarker concentrations of serum (**a**) creatinine, (**b**) cystatin C, (**c**) CK and (**d**) Total Protein at different time interval are shown [black line—No AKI (alive n = 34, death n = 1), Red line—AKIN1/2/3 (alive n = 24, death n = 5)]. The grey colour shaded area illustrates 5th–75th percentile and light grey color shaded area indicates 75th–95th percentile of the normal range based on measurements in healthy individuals.

**Figure 4:**
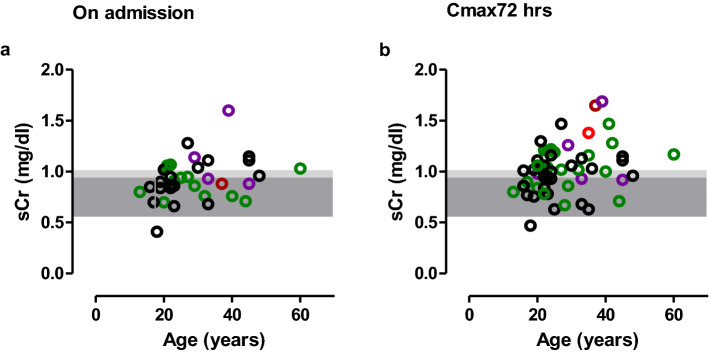
Correlation graph between age and sCr. (**a**) On admission sCr (**b**) maximum sCr concentration noted with 72 h post-ingestion (Cmax72). Black circle—No AKI (alive), red circle—death, green circle—AKIN1 (alive), purple circle—AKIN2 (alive) and brown circle AKIN3 (alive). The grey color shaded area illustrates 5th–75th percentile and light grey color shaded area indicates 75th–95th percentile of the normal range based on measurements in healthy individuals.

Serum creatinine and sCysC levels reported within 0–8 h post-ingestion and maximum sCr and sCysC observed during follow up (1–7 months post ingestion) were not significantly different among No AKI, AKIN1 and AKIN2/3 (Table 2; Fig. 3A,B). According to KDIGO criteria; 24 (40%) developed AKI (AKI1 (n = 18), AKI2 (n = 5) and AKI3 (n = 1)) and 35 (60%) did not develop AKI. Several patients in both No AKI and AKI1/2/3 groups had increased sCK concentrations. Serum cystatin C remained within the normal range (Fig. 3A,B). Patients who had the maximum sCr within 72 h were older and belonged to AKI2/3 category (Table 2). Both admission sCr (r = 0.34, p < 0.05) and maximum sCr within 72 h (r = 0.32, p < 0.05) correlated with age (Fig. 4).

**Table 2:** Demographic, laboratory, and clinical data in renal biomarker study.

Parameter	No AKI (n = 35)^#^	AKI1 (n = 18)	AKI2/3 (n = 6)
Time to admission—h (IQR)	2 (1–3)	3 (1–3)	4 (2–7)
Amount ingested (ml)	36 (11–73)	50 (9–120)	12 (6–36)
Male—n (%)	16 (46%)	14 (78%)	4 (67%)
Age in years (IQR)	23 (19–29)	26 (21–40)	35 (27–41)
GCS score at admission	15 (15–15)	15 (15–15)	15 (15–15)
Systolic BP—mmHg (IQR)	114 (110–120)	120 (110–130)	124 (100–133)
Diastolic BP—mmHg (IQR)	70 (70–80)	80 (70–80)	70 (65–80)
Heart rate—beats/minute (IQR)	81 (78–88)	80 (78–92)	80 (69–85)
Respiratory rate—per minute (IQR)	20 (16–20)	20 (16–22)	19 (18–23)
Maximum sCr change from the baseline during hospital stay (mg/dl)	0.13 (0.06–0.20)	0.33 (0.30–0.38)	0.60 (0.45–1.14)
Maximum sCr observed within 72 h (mg/dl)	0.96 (0.78–1.11)	1.03 (0.89–1.19)	1.12 (0.93–1.67)
Maximum sCr measured within 0–8 h post-ingestion (mg/dl)	0.90 (0.78–1.07)	0.93 (0.76–1.03)	0.91 (0.81–1.26)
Maximum sCr observed during follow up (mg/dl)^^^	0.71 (0.63–0.86)	0.66 (0.80–0.92)	0.84 (0.61–1.06)
Maximum sCysC observed within 72 h (mg/l)	0.81 (0.74–0.88)	0.81 (0.74–0.83)	0.79 (0.68–0.81)
Maximum sCysC measured within 0–8 h post-ingestion (mg/l)	0.78 (0.72–0.85)	0.80 (0.72–0.84)	0.68 (0.61–0.75)
Maximum sCysC observed during follow up (mg/l)^^^	0.78 (0.73–0.85)	0.79 (0.72–0.87)	1.09 (0.81–1.14)
Maximum sCK observed within 72 h (U/l)	98 (41–232)	140 (83–208)	284 (94–428)
Maximum sCK measured within 0–8 h post ingestion (U/l)	135 (62–208)	98 (13–142)	143 (52–219)
uCytoC measured within 0–8 h post ingestion (ng/mg)	0.30 (0.31–0.77)	0.51 (0.21–0.12)	1.14 (0.52–1.64)

Data expressed as number of patients (n), percentage (%) or median (lower quartile − upper quartile). ^#^There was one death. ^^^One to 7 months post-discharge.

**Figure 5:**
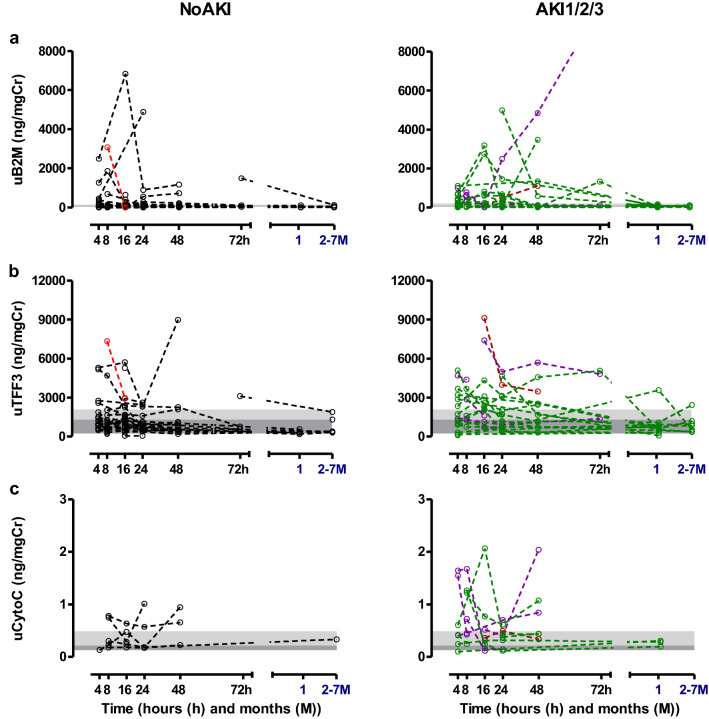
Normalized urinary biomarker profile. Individual patient’s normalized concentrations of urinary (**a**) β2M, (**b**) TFF3, (**c**) CytoC are shown [black dotted line—No AKI (alive), red dotted line—death, green dotted line—AKI1 (alive), purple dotted line—AKI2 (alive) and brown dotted line AKI3 (alive)]. The grey color shaded area illustrates 5th–75th percentile and light grey color shaded area indicates 75th–95th percentile of the normal range based on measurements in healthy individuals.

Urinary β2M, TFF3 and CytoC increased in many patients with AKI, but was also increased in 3 patients in the No AKI group within 72 h post ingestion. The peak increment of β2M, was seen between 8–48 h post ingestion while TFF3 and CytoC exhibited peaks within 24 h. All urinary biomarkers returned to normal at follow up (1–7 months). The other urinary biomarkers did not increase (Fig. 5). All urinary biomarkers performed poorly in diagnosing AKI (area under the receiver operating characteristic curve < 0.68) (Supplementary Table 1).

### Factors predicting the probability of death

**Figure 6:**
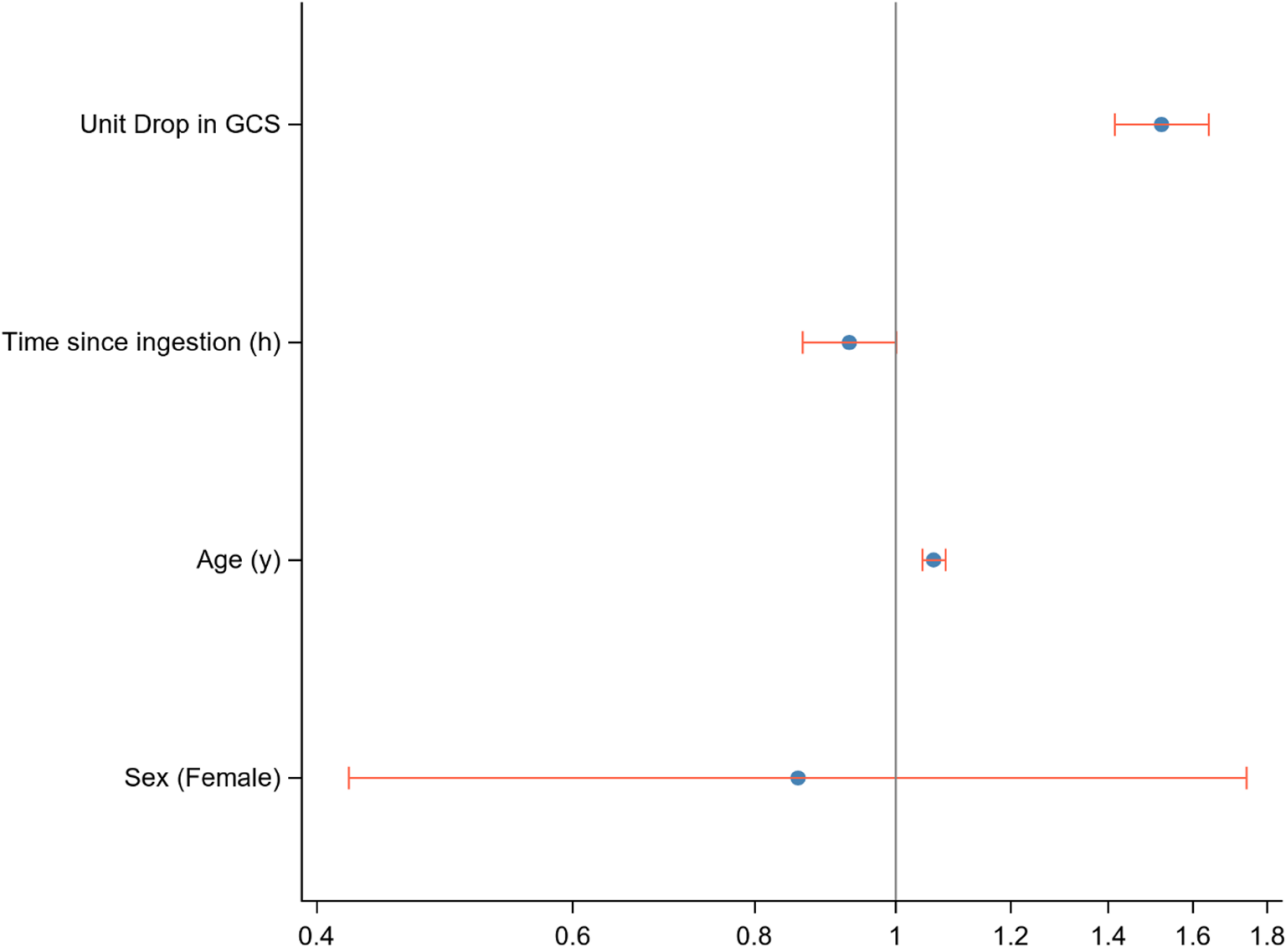
Logistic regression modelling the probability of death by sex, age, and GCS.

A logistic regression modelling the probability of death by age, sex, time since ingestion and GCS (Fig. 6) showed that sex had no influence on the likelihood of death. However, each additional year of age increased the likelihood of death by 1.06 (95% CI 1.04–1.08) and each additional drop in the GCS unit score from normal increased the likelihood of death by 1.52 (95% CI 1.41–1.64).

## Discussion

To our knowledge, this is the largest prospective case series of MCPA poisoning. Over the 18 years, there were 1653 MCPA intentional self-poisonings. Case fatality was 5.3% (95% C–P CI 4.3–6.5%) and the patients who died were generally older and had ingested larger amounts of MCPA. Severe AKI was uncommon and urinary biomarkers did not appear to aid detection.

The increased case fatality with increased age may be due to larger ingestions (perhaps due to greater suicidal intent) and/or underlying health/co-morbidity^11,12^. We have similarly noted that patients who died following ingestion of *Gloriosa superba* were older and had higher levels of plasma colchicine compared to survivors^13^.

Clinical features suggesting uncoupling of oxidative phosphorylation have been reported in MCPA poisoning cases^1,3^. Tachycardia was also identified as a key feature predicting severe poisoning and death^3^. Our study also noted significantly increased median heart rate on admission among those with a fatal outcome. These values were only marginally elevated at this time (Table 1), but death was usually delayed and further research on the progression of signs preceding death would be worthwhile.

Acute kidney injury following MCPA poisoning has been reported, however, the diagnosis in Sri Lanka is often just based on clinical observations (oliguria, dark coloured urine)^3^, with sCr laboratory confirmation only sometimes available^4^. Other conventional renal biomarkers such as blood urea nitrogen are not widely used in clinical practice in Sri Lanka^14^. Serum cystatin C may be more reliable to diagnose AKI than sCr, as muscle and/or mitochondrial toxicity may directly causes rises in sCr^15–17^. The lack of elevation in sCysC even when there was a sCr rise in MCPA poisoning (Table 2; Fig. 3A,B) suggests the rise in sCr in many cases may not be due to AKI. MCPA induces muscle toxicity^3^ (see CK levels (Fig. 3A,B)) which might explain many cases. This may explain the lack of a consistent change in AKI injury urinary biomarkers in AKI2/3 cases. Sporadic elevation of some biomarkers such as β2M, TFF3 and CytoC (Fig. 5) in patients with both AKI and No AKI might also be explained by cardiovascular or gastrointestinal toxicity^1,13,18–20^. Early increases in β2M^21^, and TFF3^22^ concentrations might also be explained by pre-renal injury, that is the reversible loss of renal function without renal structural damage.

This study provides an overview of the epidemiological data and pattern of renal injury associated with MCPA poisoning. We also identified predictors of fatal outcome. There is no specific antidote for managing MCPA poisoning, and management is largely based on supportive therapy. These predictors may be useful in triaging and allocating resources to better utilize the limited-resources available in lower-middle income countries.

## Limitations

The study hospitals included in the manuscript mainly represented 4 main provinces (of total 9) in Sri Lanka which could be a limitation when providing an overview of the MCPA poisoning in the country. However, these hospitals were either teaching, general or base hospitals which receive a larger number of transferred poisoning patients nationally and most poisoning cases are reported from this region. Furthermore, the poisoning management ward of the Teaching Hospital, Peradeniya is the only ward in Sri Lanka which is specifically designed to treat self-poisoned patients and a large number of patients from all over the country are admitted to this special unit. Therefore, the data set in the manuscript provide a reasonable representation of the MCPA poisoning in Sri Lanka.

This is by far the largest prospective cohort study to report on MCPA poisoning, however, MCPA confirmatory assays were only done on a small proportion of patients due to funding limitations. However, as reported elsewhere^5^, the history of pesticide exposure in our cohorts has always strongly correlated with subsequent assay confirmations. Although this is the most comprehensive renal biomarker study on AKI from MCPA poisoning to date, the second major limitation was that timing and completeness of biomarker sample collections was somewhat variable with missing data at some timepoints for many patients. This reflects the priority of providing patient care over research data collection, limited resources in these hospitals, and difficulty with following up patients in remote rural communities.

## Conclusion

MCPA poisoning remains a problem in Sri Lanka, predominantly in male farmers in agricultural communities, but this does not seem to be increasing despite the many bans on other herbicides. Fatal outcomes were more common in older people, partly explained by larger ingestions. Acute kidney injury was not common, and the sporadic changes in renal biomarkers may even be explained by toxicity to other organs.

## Supplementary Information

Supplementary Table 1.

## Data Availability

The original data presented in the study are included in the article/supplementary material, and further inquiries can be directed to the corresponding author.
