# Supplementary material for: Epidemiology and renal injury following 2-methyl-4-chlorophenoxyacetic acid (MCPA) poisoning
Source: Sci Rep. 2022 Dec 19;12:21940. doi: 10.1038/s41598-022-25313-z (PMC9763389; doi:10.1038/s41598-022-25313-z)
Supplement: Supplementary file 1 — Supplementary Table 1. [file 41598_2022_25313_MOESM1_ESM.docx]

**Epidemiology and renal injury following 2-Methyl-4-chlorophenoxyacetic acid (MCPA) poisoning**

Thilini M Wijerathna^1,2*^, Nicholas A Buckley^1,3^, Indika B Gawarammana^1,4^, Jacques Raubenheimer^3^, Seyed Shahmy^1,5^, Umesh Chathuranga^1^, Chathura Palangasinghe^1^, Fathima Shihana^1,3^, Fahim Mohamed^1,6,7,8^

^1^South Asian Clinical Toxicology Research Collaboration, Faculty of Medicine, University of Peradeniya, Peradeniya, Sri Lanka

^2^Department of Biosystems Technology, Faculty of Technology, General Sir John Kotelawala Defence University, Ratmalana, Sri Lanka

^3^The University of Sydney, Faculty of Medicine and Health, Biomedical informatics and Digital Health, Clinical Pharmacology and Toxicology Research Group, Sydney, NSW, Australia 2006

^4^Department of Medicine, Faculty of Medicine, University of Peradeniya, Peradeniya, Sri Lanka

^5^National Science and Technology Commission of Sri Lanka, Colombo, Sri Lanka

^6^Department of Pharmacy, Faculty of Allied Health Science, University of Peradeniya, Peradeniya, Sri Lanka

^7^National Poison Centre, University Sains Malaysia, Penang Malaysia

^8^Australian Kidney Biomarker Reference Laboratory, Department of Nephrology, Prince of Wales Hospital and Clinical School, University of New South Wales, Sydney, Australia

**Corresponding Author**

Thilini M Wijerathna

South Asian Clinical Toxicology Research Collaboration, Faculty of Medicine, University of Peradeniya, Peradeniya, Sri Lanka

Department of Biosystems Technology, Faculty of Technology, General Sir John Kotelawala Defence University, Ratmalana, Sri Lanka

**Email**

wijerathnapa@kdu.ac.lk

**Supplementary Tables**

**Supplementary Table 1 Area under the ROC (AUC-ROC) curve values for normalized urinary biomarkers**

| Biomarker | AUC-ROC | 95% CI | Performances |
| --- | --- | --- | --- |
| KIM-1 | 0.50 | 0.32 to 0.68 | Fail |
| Clusterin | 0.58 | 0.40 to 0.75 | Fail |
| Albumin | 0.60 | 0.44 to 0.75 | Poor |
| β2M* | 0.68 | 0.53 to 0.83 | Poor |
| CysC* | 0.66 | 0.51 to 0.81 | Poor |
| NAGL | 0.57 | 0.41 to 0.72 | Fail |
| OPN | 0.57 | 0.41 to 0.72 | Poor |
| TFF3* | 0.66 | 0.50 to 0.81 | Poor |
| CytoC | 0.66 | 0.38 to 0.94 | Poor |

AUC-ROC values were computed between NoAKI and NoAKI groups. * Statistically significant (p < 0.005). AUC ≥ 0.9 Excellent, 0.8 ≤ AUC < 0.9 Good, 0.7 ≤ AUC < 0.8 Fair/Moderate, 0.6 ≤ AUC < 0.7 Poor, AUC ≤ 0.6 – Fail[1]

References

1. Nahm, F.S., *Receiver operating characteristic curve: overview and practical use for clinicians.* Korean J Anesthesiol, 2022. **75**(1): p. 25-36.
